# von Willebrand Factor, Free Hemoglobin and Thrombosis in ECMO

**DOI:** 10.3389/fmed.2018.00228

**Published:** 2018-08-17

**Authors:** Christian Valladolid, Andrew Yee, Miguel A. Cruz

**Affiliations:** ^1^Molecular Physiology and Biophysics, Baylor College of Medicine, Houston, TX, United States; ^2^Center for Translational Research on Inflammatory Diseases, Michael E. DeBakey VA Medical Center, Houston, TX, United States; ^3^Pediatrics-Hematology, Baylor College of Medicine, Houston, TX, United States; ^4^Department of Medicine, Baylor College of Medicine, Houston, TX, United States

**Keywords:** ECMO induced coagulopathy, VWF, free hemoglobin, thrombosis, fibrinogen

## Abstract

Though extracorporeal membrane oxygenation (ECMO) provides life-saving support, this intervention exposes patients to certain risks. Circulating free hemoglobin (fHb) resulting from mechanically induced hemolysis and insufficient haptoglobin/hemopexin may promote thrombosis within the ECMO circuit. Thrombi in the circuit can result in thromboembolic complications in these patients. Prevention of thrombus formation and propagation in the ECMO circuit may improve clinical outcome. fHb released during hemolysis has been shown to have multiple adverse effects, including thrombosis, but the mechanism by which fHb contributes to thrombosis in an ECMO circuit remains elusive. It is well established that (1) high shear stress generated in the circuit may cause hemolysis, and (2) plasma fibrinogen is adsorbed onto the inner tubing of the ECMO circuit over time. Plasma von Willebrand factor (pVWF) mediates platelet deposition at sites of vascular injury under high shear stress by sensing alterations in the hemodynamic environment. This biophysical property of pVWF that enables hemostasis may also contribute to the pathogenesis of ECMO-induced thrombosis. pVWF contains binding sites for both adsorbed fibrin(ogen) and fHb. High concentrations of fHb increase pVWF-mediated platelet adhesion and thrombus formation on a surface-adsorbed fibrin(ogen) under high shear stress. The molecular mechanism(s) by which fHb drives the conformation of pVWF into a prothrombotic state is currently unknown. Reduction of thrombotic risks during ECMO intervention warrants further investigations into the interaction between pVWF and fHb.

## Background

Extracorporeal membrane oxygenation (ECMO) provides temporary multi-organ support for patients with congenital or acquired heart and/or lung disease or resuscitative support for patients with cardiopulmonary failure. ECMO functionally substitutes the heart and lungs by circulating blood through a gas exchanger similarly to a heart-lung bypass machine but for a longer duration. Patients supported with ECMO can present with both bleeding and thromboembolic risks despite close monitoring of systemic anticoagulation (1). Bleeding complications may arise due to multiple reasons. Thromboembolic risks stem from procoagulant surfaces and complex hemodynamic environments within the ECMO circuitry despite the use of markedly improved biocompatible materials (1). Additionally, hemolysis associated with pathological hemodynamics within the ECMO circuit may contribute to systemic dysregulation of hemostasis (1–3).

Increasing evidence suggests elevated levels of free hemoglobin (fHb) in blood of patients on ECMO are a major clinical complication (3, 4). High concentrations of fHb are cytotoxic and associated with an increased incidence of thrombosis, morbidity, and mortality in patients on ECMO (5–8). Prevention of circuit clotting in ECMO may improve clinical outcome.

von Willebrand factor (VWF) is a multimeric glycoprotein that plays a critical function in mediating platelet adhesion and aggregation in hemostasis and thrombosis (9). The mature subunit of VWF contains domains that are arranged in the order D′D3-A1-A2-A3-D4-(C1-C6)-CK (10). The triplicate A domains (A1-A2-A3) in the central portion of the VWF subunit contains essential binding sites for the biology of initiating platelet recruitment to sites of vascular injury. The A1 domain binds to platelet glycoprotein (GP)Ibα; the metalloprotease, ADAMTS-13, cleaves multimeric VWF within the A2 domain to regulate VWF's size and thus, hemostatic capacity; and the A3 domain binds to collagen (11–13).

Normally, plasma (p)VWF does not interact with circulating platelets unless it undergoes conformational changes. In hemostasis, when the integrity of the vessel wall has been compromised, pVWF quickly binds to components exposed in the subendothelial matrix (e.g., collagen) at high shear stress. This interaction provokes structural changes that expose the contact site for the platelet receptor GPIbα in A1 domain of VWF, allowing circulating platelets to rapidly bind to the collagen-bound VWF (14, 15). On the other hand, pVWF can also mediate platelet adhesion/aggregation in the absence of an evident physical damage in the vasculature under pathological shear stress. It is most likely that an elevated shear rate within a pathological hydrodynamic environment exposes pVWF to greater tension that unfolds the triplicate A1-A2-A3 domains, disrupts the autoinhibitory mechanism formed by the interaction between A1 and A2 domains, and thus, increases the VWF-GPIbα binding (16–20). Conformational changes in pVWF that promotes GPIbα binding can also be biochemically induced with the antibiotic, ristocetin (21). This compound makes direct contacts to amino acid sequences in the flanking regions of the A1 domain structure and promotes the exposure of ligand binding sites in both A1 and A2 domains (22).

## Effect of the binding of free hemoglobin to VWF

We and others have demonstrated the capacity of fHb to block the cleavage of VWF by ADAMTS-13 (23, 24). Some clinical studies have indicated that a reduction of VWF cleavage by high levels of fHb causes thrombosis in circulatory devices (25, 26). An explanation for this inhibitory effect could be that suprahydrodynamic forces unfold and activate pVWF (exposed A1A2 domains), allowing fHb to gain access to the A2 domain of VWF and competitively block ADAMTS-13 mediated proteolysis. Consequently, activated pVWF bind to platelets, leading to platelet aggregation and thrombus formation.

On the other hand, we speculate that fHb causes thrombosis on surfaces with adsorbed fibrinogen (like the inner tubing of an ECMO circuit) by strengthening the interaction between VWF and GPIbα. The concept is based on our identification and characterization of a novel binding activity of fHb for the A1 domain of VWF and how this interaction seems to be associated with high levels of fHb and thrombosis in patients on ECMO (27). fHb binds to the VWF A1 domain with a binding constant of ~15 μM and increases platelet deposition to fibrin(ogen)-coated surface with a threshold level of ≥ 50 mg/dL (~30 μM heme), a concentration that favors the formation of the fHb-A1 complex in circulation. Importantly, clinical evidence indicates that the incidence of thrombosis in patients on mechanical circulatory devices correlates with levels of fHb ≥ 50 mg/dL (5). Additionally, fHb significantly increases the arrest of circulating platelets to a surface coated with purified recombinant triple A1-A2-A3 domains of VWF at high shear stress (27). Antibodies against GPIbα or VWF deficiency blocks the enhancement of platelet adhesion by fHb, validating the importance of VWF (i.e., A1 domain) to mediate thrombus formation in the presence of high levels of fHb (27).

The adsorption of plasma fibrinogen to biomaterials is a quick event that transforms the surface to one that promotes platelet adhesion and thrombus formation under high shear stress as seen in ECMO circuits (28). It is well appreciated that platelet adhesion to fibrin(ogen)-coated surface is mainly mediated by pVWF at high shear stress (29, 30). To date, a number of laboratories are focused on creating biomaterials capable of reducing the adsorption of fibrinogen and consequently reducing the risk of thrombosis [reviewed in Jaffer et al. (31)]. On the other hand, the conformation of surface-adsorbed fibrin(ogen) remains unknown, and this missing information delays the development of inhibitors targeting the adsorbed fibrin(ogen) to block the interaction with pVWF. Furthermore, the mechanism(s) by which pVWF interacts with adsorbed fibrin(ogen) has not been investigated regarding thrombosis within an ECMO circuit. Limited studies have examined the interaction between VWF and fibrin(ogen), but the identity of the interface(s) remains unclear. Keuren et al. indicated that VWF residues distal of the triplicate A1-A2-A3 domains form the interface with fibrin (32). Studies from our laboratory have also demonstrated the binding of pVWF to adsorbed fibrin(ogen) in a ristocetin-dependent manner (33), suggesting that the antibiotic may expose novel cryptic-binding sites for fibrin(ogen) within the A1A2 domains of VWF (22). Further dissection of the VWF A domains found binding activity in the A2 domain for fibrin(ogen) (33), and very recently, we observed that its homologous A1 domain also has binding activity for fibrin(ogen) and effectively mediates firm platelet adhesion at high shear stress (ongoing investigation). We speculate that, like ristocetin, an interaction between fHb and A1A2 domains possibly induces the binding of pVWF to a fibrin(ogen)-coated surface. Under supraphysiological shear stress (i.e., ECMO), the immobilized VWF-fHb complex would rapidly capture platelets and lead to thrombosis. In fact, we have shown that whole blood perfused with fHb over fibrin(ogen)-coated surfaces markedly increased platelet deposition and thrombus formation as compared to blood perfused without fHb at high shear stress (27).

Finally, we previously demonstrated that *in vitro* heparin had a weak inhibitory effect in reducing thrombosis in the presence of high levels of fHb (27). This result could explain the formation of clots in the circuit even though heparin is continuously infused in patients on ECMO. Thus, in addition to developing a better coating for biomaterials to prevent fibrinogen adsorption or inhibitors for the binding of VWF to adsorbed fibrin(ogen), targeting the fHb-VWF appears to be another option to reduce thrombosis in the ECMO circuit.

In summary, clinical data support a direct correlation between high levels of fHb and thrombosis in patients on mechanical circulatory devices. There is no doubt of the need for more research on the molecular mechanisms involved in the interaction of fHb with VWF as this interaction may have important ramifications in the etiology and treatment of other hemolytic disorders. fHb possibly makes pVWF more prothrombotic by blocking the cleavage of the hypercoagulable high shear stress-activated VWF multimers, and by increasing the binding of pVWF to platelet GPIbα and immobilized fibrin(ogen). The more insights we gain from dissecting the molecular mechanisms by which fHb promotes thrombosis via VWF will lay the foundation for the development of potential therapies to prevent fHb-VWF binding and improve clinical outcome.

## Author contributions

MC, AY, and CV wrote this article. MC and CV designed and performed experiments.

### Conflict of interest statement

The authors declare that the research was conducted in the absence of any commercial or financial relationships that could be construed as a potential conflict of interest.
